# Novel Anti-Acetylcholinesterase Effect of *Euonymus laxiflorus* Champ. Extracts via Experimental and In Silico Studies

**DOI:** 10.3390/life13061281

**Published:** 2023-05-30

**Authors:** Van Bon Nguyen, San-Lang Wang, Tu Quy Phan, Manh Dung Doan, Thi Kim Phung Phan, Thi Kim Thu Phan, Thi Huyen Thoa Pham, Anh Dzung Nguyen

**Affiliations:** 1 Institute of Biotechnology and Environment, Tay Nguyen University, Buon Ma Thuot 630000, Vietnam; dmdung@ttn.edu.vn (M.D.D.); nadzung@ttn.edu.vn (A.D.N.); 2 Department of Chemistry, Tamkang University, New Taipei City 25137, Taiwan; 3 Life Science Development Center, Tamkang University, New Taipei City 25137, Taiwan; 4 Faculty of Medicine and Pharmacy, Tay Nguyen University, Buon Ma Thuot 630000, Vietnam; phantuquy@ttn.edu.vn (T.Q.P.); ptkphung@ttn.edu.vn (T.K.P.P.); 5 Department of Science and Technology, Tay Nguyen University, Buon Ma Thuot 630000, Vietnam; ptkthu@ttn.edu.vn (T.K.T.P.); pththoa@ttn.edu.vn (T.H.T.P.)

**Keywords:** Alzheimer, acetylcholinesterase inhibitors, *Euonymus laxiflorus* Champ., bioactive compounds, medicinal plants

## Abstract

Alzheimer’s disease (AD) is the most common form of dementia, which is recorded as a global health issue. Natural acetylcholinesterase inhibitors (AChEIs) are considered a helpful therapy for the management of symptoms of patients with mild-to-moderate AD. This work aimed to investigate and characterize *Euonymus laxiflorus* Champ. (ELC) as a natural source of AChEIs compounds via in vitro and virtual studies. The screening parts used, including the leaves, heartwood, and trunk bark of ELC, revealed that the trunk bark extract possessed the highest activity, phenolics and flavonoid content. The in vitro anti-Alzheimer activity of ELC trunk bark was notably reclaimed for the first time with comparable effect (IC_50_ = 0.332 mg/mL) as that of a commercial AChEI, berberine chloride (IC_50_ = 0.314 mg/mL). Among various solvents, methanol was the most suitable to extract ELC trunk bark with the highest activity. Twenty-one secondary metabolites (**1**–**21**) were identified from ELC trunk bark extract, based on GCMS and UHPLC analyses. Of these, 10 volatile compounds were identified from this herbal extract for the first time. One phenolic (**11**) and seven flavonoid compounds (**15**–**21**) were also newly found in this herbal extract. Of the identified compounds, chlorogenic acid (**11**), epigallocatechin gallate (**12**), epicatechin (**13**), apigetrin (**18**), and quercetin (**20**) were major compounds with a significant content of 395.8–2481.5 μg/g of dried extract. According to docking-based simulation, compounds (**11**–**19**, and **21**) demonstrated more effective inhibitory activity than berberine chloride, with good binding energy (DS values: −12.3 to −14.4 kcal/mol) and acceptable RMSD values (0.77–1.75 Å). In general, these identified compounds processed drug properties and were non-toxic for human use, based on Lipinski’s rule of five and ADMET analyses.

## 1. Introduction

Alzheimer’s disease (AD) is the most common form of dementia. This disease has been recorded as a global health issue, with the number of patients worldwide reaching approximately 50 million; the number of cases of AD may double every 5 years, and it is estimated to reach 152 million by 2050 [1,2]. AD significantly affects individuals, the families of the patients, and the global economy, with the annual incurred costs estimated to reach about USD 1 trillion worldwide [1]. Up to date, no therapies may completely cure AD. However, some current therapies may significantly help to manage the symptoms, provide temporary relief, and increase the quality of life of patients with AD [3]. Among the therapies, the utilization of herbs for the management of AD has been considered since they are natural sources of bioactive compounds and are less toxic [4,5,6,7,8,9,10].

Vietnam, a tropical country, has been ranked as the sixteenth most biodiverse worldwide, with more than 10,000 herbal species recorded and 4000 species in use as traditional medicine [11]. Thus, studies based on the investigation of the biological effects and chemical profiles of medicinal plants from Vietnam have received increasing interest in recent years [12,13,14,15,16,17]. However, only a few studies and new records of herbs with potential anti-Alzheimer effects of this biodiverse area have been reported [15,16,17]; as such, the discovery of medicinal plants with biological activities related to Alzheimer’s drugs from Vietnam has been received with great interest.

*Euonymus laxiflorus* Champ. (ELC), a medicinal plant, is wildly grown in Vietnam, India, Cambodia, Myanmar, and China [12,14,18]. ELC has been found to be a rich source of bioactivities, including antioxidant and anti-nitric oxide (anti-NO) properties [19], a potent anti-enzyme targeting anti-diabetes, such as anti-α-glucosidase and anti-α-amylase [12,14], and a significant reduction in the blood glucose of normal and diabetic rats with fewer or no side effects [20,21]. Some target compounds of α-glucosidase, α-amylase, antioxidant, and the anti-NO effect were purified and identified in our earlier works [13,19,22]. However, no data are available on anti-acetylcholinesterase (AChE) targeting anti-Alzheimer, as well as the concerning constituents of this herb.

**Scheme 1:**
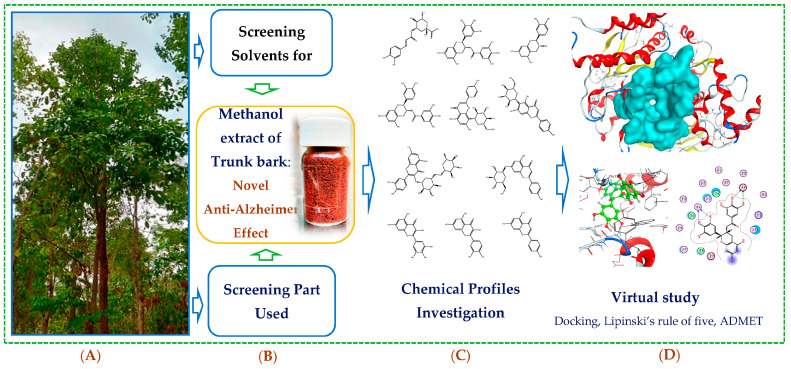
A schematic of this study. (**A**) The samples of *Euonymus laxiflorus* Champ. (**B**) were collected for screening extraction solvents and functional part used. (**C**) The most bioactive extract was investigated for its chemical profiles, (**D**) then the virtual study was conducted for the prediction of active compounds and drug discovery.

As a part of our ongoing objective to investigate novel bioactivity and the active constituents of the herbal ELC for the development of this herb as a natural and functional food and/or as drugs, in this investigation, we collected this herb from Dak Lak province of Vietnam and determined the bioactivities. This study is the first to report the anti-AChE activity of ELC. Further screening of the most active part used, including the heartwood, trunk bark, leaves of ELC, and the chemical profiles, the interaction of active molecules toward the targeting enzyme acetylcholinesterase, as well as Lipinski’s rule of five and ADMET-based pharmacokinetics and pharmacology analyses via computational study are also presented in this study. All steps of our work are summarized in Scheme 1.

## 2. Materials and Methods

### 2.1. Materials

The samples of some parts used, such as leaves, heartwood, and trunk bark of ELC, were collected in Yok Don National Park, Dak Lak Province, Vietnam, in 2022. The dried herbal parts were packed in PE bags and stored at −30 °C before extraction. Acetylcholinesterase and berberine chloride were acquired from Sigma Aldrich (St. Louis, MO, USA). The highest-grade solvents and common chemical agents available were used in this study.

### 2.2. Chemical Methods

#### 2.2.1. Preparation of Herbal Extracts

Herbal extracts were prepared using the previously reported process [12,13]. The herbal samples were extracted using different solvents, including methanol, butanol, ethyl acetate, *n*-hexane, and water. The solvent was mixed with the plant sample for 24 h in a ratio of 100 mL/10 g in a conical glass flask using a shaker; then, the solution was filtered via a filter paper (no. 1, Whatman International Ltd., Maidstone, UK) at room temperature. The sample residues were further extracted twice with 200 mL using the same solvent with the same conditions described above. These three solutions were combined and concentrated at 60 °C under a vacuum using a rotary evaporator (IKA, Staufen, Germany). The extracts were stored at −30 °C before further use [12,13].

#### 2.2.2. Gas Chromatography-Mass Spectrometry (GCMS) Analysis

The herbal extract was dissolved in methanol (MeOH), then purified by solid-phase extraction using the QuEChERS method. For further analysis, GC (Thermo Trace GC Ultra, USA) and ITQ900 (Thermo, Waltham, MA, USA) were conducted. A TG-SQC capillary column (30 m × 0.25 mm × 0.25 μm) was utilized for the GCMS analysis. Helium (99.999%), a carrier gas, was set at a constant flow rate of 1 mL/min. The sample solution (1 μL) was injected in a split ratio of 10:1. The temperature of the injector and the ion source were set at 250 and 230 °C, respectively. The temperature program of the oven was set at 70 °C (isothermal for 2 min) and increased up to 280 °C with an increasing speed of 15 °C/min, ending with a 10 min isothermal at 280 °C. MS data were at 70 eV, a scanning interval time of 0.5 s, and for fragments from 50 to 650 Da. The compounds were identified via comparison with reported compounds using compounds data of the Mass Spectra Library (NIST 17.L and Wiley).

#### 2.2.3. UHPLC Analysis

The herbal extracts were dissolved in MeOH at 10 mg/mL and then filtered using a 0.45 μm polyvinylidene fluoride membrane filter (Millipore Sigma, Billerica, MA, USA). An amount of two microliters of the extract solution was injected into the UHPLC system (Thermo Ultimate 3000). The constituents in the sample were separated via a column (Hypersil GOLD aQ, 3 μm, 150 × 2.1 mm), which was maintained at a temperature of 30 ℃. A mobile phase consisting of MeOH and 0.1% phosphoric acid in water was used, and the mobile phase program was set at 5% MeOH (0.0–0.5 min), 5–30% MeOH (0.5–8.0 min), 30–45% MeOH (8.0–13 min), 45–65% MeOH (13.0–18.0 min), 65–95% MeOH (18.0–22.0 min), and 95–5% MeOH (22.0–23.0 min). The flow was set at 0.2 mL/min, and the constituents were detected at 265 nm.

### 2.3. Enzyme Inhibition Assay

AChE was used to carry out the inhibitory assay using Ellman’s method [23] with modifications. A 0.05 M phosphate buffer, pH 8.0, was used in this protocol. Additionally, 60 µL of the herbal extract was mixed with the same volume of 0.5 mM enzyme solution and 120 µL of phosphate buffer, and kept at 25 °C for 15 min in a flat-bottom 96-well plate. Agent 5, 5′-dithiobis-2-nitrobenzoic acid (DTNB, 30 µL of 0.003 M) and 40 µL of 0.002 M acetylthiocholine iodide (ATCI) were added into the mixture to start the reaction. The reaction was maintained at 25 °C for 10 min before the absorbance was measured at the wavelength of 415 nm. For the control group, 60 µL of phosphate buffer was used instead of 60 µL of the herbal extract solution and the same condition was used for the calculation of enzyme inhibition activity using the following equation:

AChE inhibition (%) = (A_C_ − A_E_)/A_C_ × 100,

where A_E_ is the absorbance value measured at 415 nm of the reaction containing herbal extract and enzyme, while A_C_ is the absorbance value at 415 nm of the reaction containing enzyme and phosphate buffer instead of the herbal extract. The samples and berberine chloride (commercial AChE inhibitor) were dissolved in dimethylsulfoxide and eluted at various concentrations using a 0.05 M phosphate buffer and then used for the tests.

### 2.4. Virtual Study Methods

#### 2.4.1. Docking Simulation

Virtual analysis was conducted using the MOE-2015.10 software to predict the active metabolite-concerning inhibition against the targeting enzyme. This virtual protocol was performed following the three typical steps mentioned in the previous reports [24,25].

✓ Preparation of protein structure: The structure data of enzyme AChE were obtained from the Research Collaboratory for Structural Bioinformatics Protein Data Bank for the preparation of their three-dimensional structures through the use of MOE-2015.10. The most active sites on the enzyme were found using the site finder function of the MOE software. A virtual pH 7 was used for the preparation of enzyme structure.
✓ Preparation of ligand structures: The structures of identified compounds (ligands) from the herbal extracts and commercial inhibitors were prepared using the ChemBioOffice 2018 software and further optimized using the MOE software. The following parameters were required to prepare the structure of ligands: virtual pH 7, force field MMFF94x; R-Field 1:80; cutoff, rigid water molecules, space group p1, cell size 10, 10, 10; cell shape 90, 90, 90; gradient 0.01 RMS kcal·mol^−1^A^−2^.
✓ Docking ligands into enzymes and the obtained out-put data: The prepared ligands were docked into the active site of AChE using the MOE software. As major out-put for analysis, DS value, RMSD value, linkage types, compositions of amino acids, and the linkages’ distances were harvested.

#### 2.4.2. The Lipinski Rule of Five and ADMET Analyses

The online software accessed at (http://www.scfbioiitd.res.in/software/drugdesign/lipinski.jsp (accessed on 15 May 2023)) was utilized for the Lipinski Rule of Five performance. A web tool called SwissADME (http://www.swissadme.ch/ (accessed on 15 May 2023)) was used for the prediction of some pharmacokinetic parameters of ADMET. The out-put data of theoretical interpretations of pharmacokinetic parameters have been previously described [26] and used as a public reference online, accessed at (http://biosig.unimelb.edu.au/pkcsm/theory (accessed on 15 May 2023)).

## 3. Results and Discussion

### 3.1. New Records of the Potential Anti-Acetylcholinesterase Effect of Euonymus laxiflorus Champ

**Figure A1:**
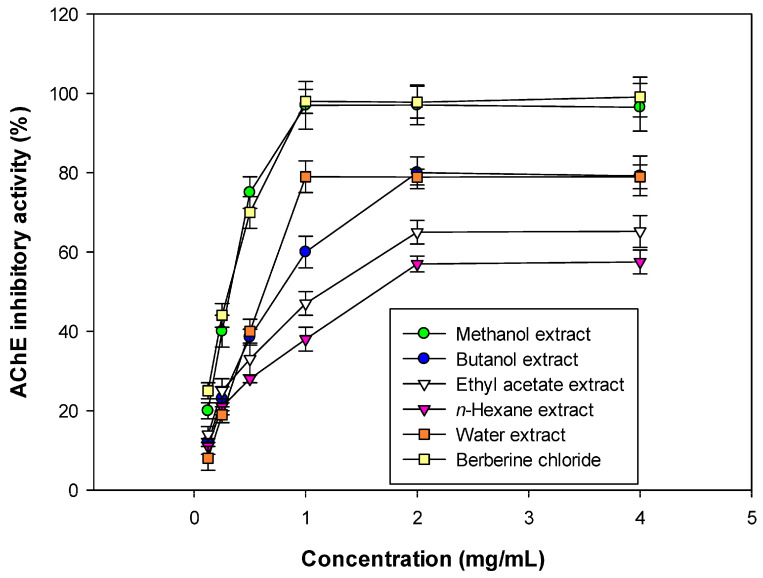
AChE inhibitory activity (%) of *Euonymus laxiflorus* Champ. trunk bark extracted by various.

To evaluate ELC as a source of anti-Alzheimer drugs, the ELC trunk bark (ELCTB) was extracted with methanol, butanol, ethyl acetate, *n*-hexane, and water, and then tested for their inhibition against AChE, a key enzyme in the discovery of anti-Alzheimer drugs [27]. The biological effect was recorded and shown under the IC_50_ value, which is the sample concentration that reduces 50% of enzymatic effect; the lower the value a sample yields, the greater the effect it displays. The results are shown in Table 1, and Figure A1 shows that the MeOH extract of ELCTB could most efficiently inhibit AChE with a low IC_50_ value (0.323 mg/mL), comparable to that of a commercial inhibitor compound, berberine chloride (IC_50_ = 0.314 mg/mL). This meOH extract was also found to inhibit AChE with a high max inhibition value of 97.0%, while other extracts showed a max effect of less than 80.1%.

**Figure A2:**
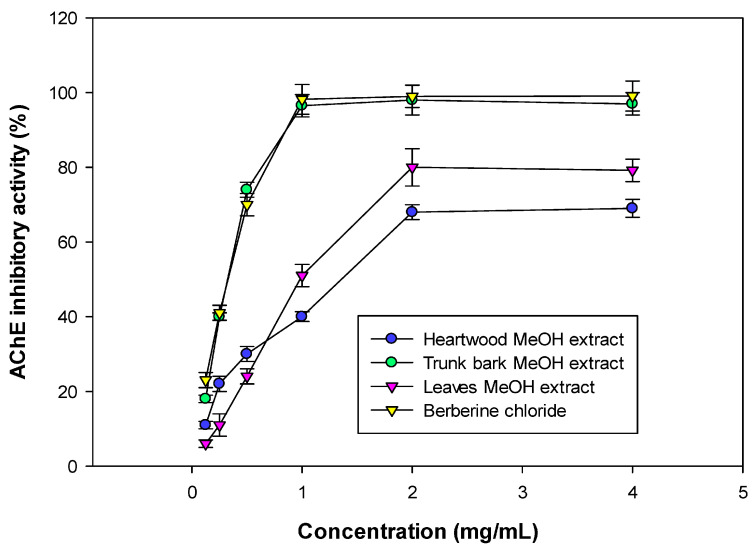
AChE inhibitory activity (%) of different parts of *Euonymus laxiflorus* Champ.

To screen for the most functional part to be used, the heartwood, trunk bark, and leaves of ELC were also extracted by meOH and used for the activity tests. The trunk bark MeOH extract showed the most AChE inhibitory activity with the smallest IC_50_ values of 0.336 mg/mL (Table 2 and Figure A2). This part used was also rich in total content of polyphenol (567.3 GAE/g dry extracts) and flavonoid (335.2 QE/g dry extracts). Thus, the MeOH extract of the trunk bark was further examined.

Recently, ELCTB has been found showing several medical effects, including antioxidant, anti-NO, anti-α-glucosidase, anti-α-amylase, and antidiabetic effects, and ELCTB has also been used by folks for the treatment of several diseases [12,14,19,20,21]. However, the potential AChE inhibitory effect of ELC was newly recorded in this study; as such, the findings herein contributed to enriching the catalog of medical effects of this plant.

Clinical trials using herb extracts in AD treatment were conducted in some works. Some herbal extracts in China achieved good effects in clinical trials for treating dementia [28]. *Ginkgo biloba* extracts were trialed widely in AD treatment and trials were conducted for almost 3–6 months [29]. In the Ginkgo One Tablet A Day (GOTADAY) trial, the dose of the one-daily drug containing 240 mg of *G. biloba* extract showed a significant improvement in neuropsychiatric symptoms and cognition for dementia patients [30]. Overall, other reports of this herb showed non-potential or unreliable effects in trials [29]. Furthermore, a patient group using a high dose of *Panax ginseng* recorded significant results based on clinical dementia rating and Alzheimer disease assessment scale when compared with the control [31]. In Iran, *Salvia officinalis* extract was tested with a fixed dose of 60 drops/day for mild-to-moderate Alzheimer’s patients in a 4-month period. It supported improved cognition after 16 weeks of treatment; however, some of its side effects need further confirmation [32]. Based on some of the above literature, using herb extracts as AD treatment agents is also considered a promising orientation. ELC extracts can also be suggested as a potential source for in-depth trials. This herb was demonstrated non-toxic for normal cells and mouse models and it has also been used as a traditional medicine by ethnic minorities in Vietnam for a long time [22].

### 3.2. The Chemical Profile of the MeOH Extract of Euonymus laxiflorus Champ. Trunk Bark

**Figure 1:**
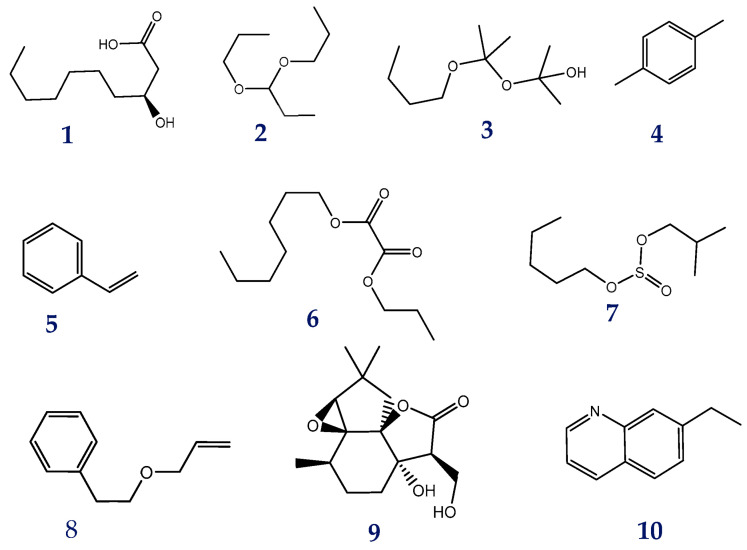
The volatile compounds detected from the methanol extract of ELC trunk bark via GCMS analysis.

**Figure A3:**
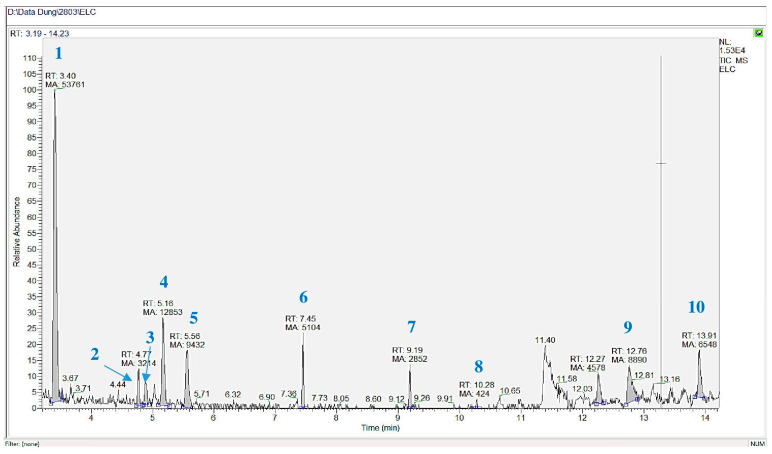
GC profile volatile compounds identified from the MeOH extract of *Euonymus laxiflorus* Champ. trunk bark.

GCMS and UHPLC analyses were used for the identification of the chemical profile of ELCTB MeOH extract. Based on GCMS analysis using compounds data from the MS Library (NIST 17.L and Wiley), 10 volatile compounds (symbolized as **1**–**10**) in ELCTB were detected and identified (Figure 1, Table 3), including 3-hydroxydecanoic acid (**1**) [33], propane, 1,1-dipropoxy- (CAS) (**2**), (2-(2-butoxyisopropoxy)-2-isopropanol (**3**), p-xylene (**4**) [34], styrene (**5**) [35], oxalic acid, heptyl propyl ester (**6**) [36], sulfurous acid, isobutyl pentyl ester (**7**) [37], 2-phenylethyl allyl ether (**8**), 12-hydroxyalliacolide (**9**) [38], and 7-ethyl-quinoline (**10**). Of these volatile compounds, 3-hydroxydecanoic acid (**1**) was found as a major volatile compound in ELCTB extract in a significantly high amount (48.63% area), followed by p-xylene (**4**), styrene (**5**), oxalic acid, heptyl propyl ester (**6**), 12-hydroxyalliacolide (**9**), and 7-ethyl-quinoline (**10**) with recoded area in the range of 4.14–11.63%; other volatiles were present in a minor amount (0.38–2.91% area). The GC profiles of these volatile compounds are presented in Figure A3.

**Figure 2:**
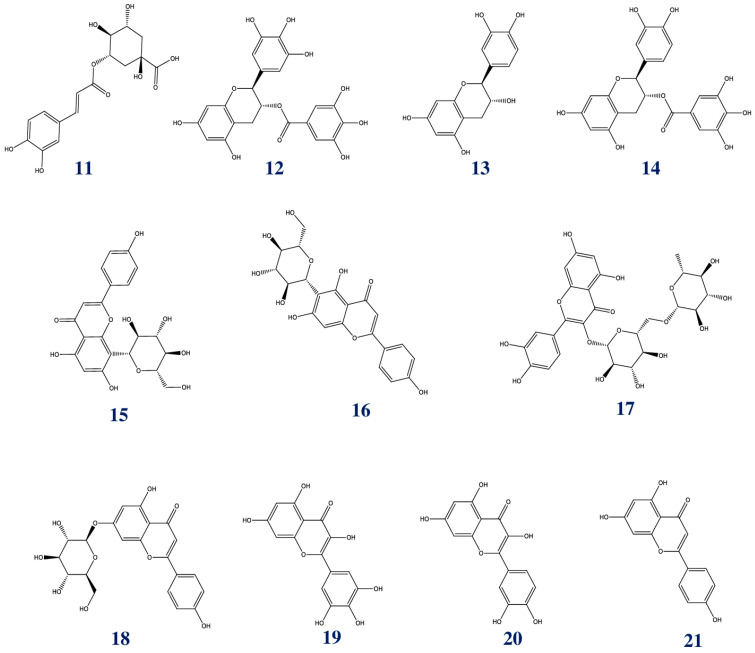
Flavonoid and phenolic compounds detected from the methanol extract of ELC trunk bark by UPLC.

**Figure A4:**
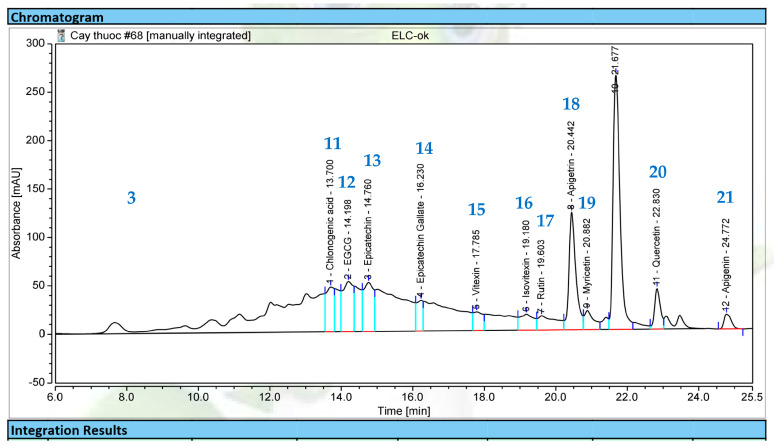
High-performance liquid chromatography finger printings of the MeOH extract of *Euonymus laxiflorus* Champ. trunk bark.

Based on the application of UHPLC using commercial compounds as standards, one phenolic (symbolized as **11**) and ten flavonoid compounds (symbolized as **12**–**21**) were detected and identified (Figure 2), including chlorogenic acid (**11**) [39], epigallocatechin gallate (EGCG) (**12**) [40], epicatechin (**13**) [41], epicatechin gallate (**14**) [42], vitexin (**15**) [43], isovitexin (**16**) [44], rutin (**17**) [45], apigetrin (**18**) [46], myricetin (**19**) [47], quercetin (**20**) [48], and apigenin (**21**) [49]. The contents of these compounds were determined and are presented in Table 4. Among these compounds, a high yield of apigetrin (**18**) was found in the ELCTB extract at 2481.525 μg/g of dried extract. Chlorogenic acid (**11**), EGCG (**12**), epicatechin (**13**), and quercetin (**20**) were detected with a significant content of 395.808–576.809 μg/g of dried extract, while the content of other compounds, **14**–**17** and **21** was lower at 23.197–184.798 μg/g of dried extract. The UHPLC profiles of these identified compounds are presented in Figure A4.

Regarding the chemical profiles of ELC, a total of 36 compounds were previously purified and their chemical structures elucidated [13,19,21,22,27,50]. Of these, six compounds were first purified in ELC stems and leaves collected from Taiwan by Kuo et al. 2003 [50]. Recently, we isolated and identified chemical structures of 30 phenolics from the methanol extract of ELC trunk bark collected from the Central Highland of Vietnam [13,19,21,22,27]. However, there are no data on volatile compounds contained in the ELCTB identified through GCMS analysis so far. Thus, this work is the first to report the application of UHPLC in the detection and determination of major phenolic and flavonoid compounds contained in the extract of this herbal species. One phenolic compound, chlorogenic acid (**11**), and seven flavonoids including vitexin (**15**), isovitexin (**16**), rutin (**17**), apigetrin (**18**), myricetin (**19**), quercetin (**20**), and apigenin (**21**) in ELC extract are reported for the first time. Thus, the experimental data of this work contributed to enriching the chemical profiles of ELC.

### 3.3. Insight into the Interactions and Energy Binding Bioactive Compounds toward Targeting Enzyme—Acetylcholinesterase via Docking Study

**Figure 3:**
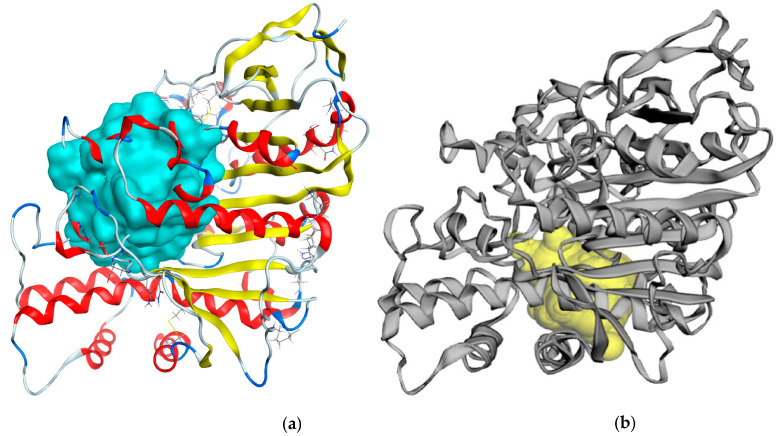
The 3D structure of the most active binding site 1 of acetylcholinesterase (**a**) was found using MOE-2015.10, and the 3D structure of the catalytic site of acetylcholinesterase (**b**) found by utilization of the CASTp3.0.

The active compounds, the interaction and binding energy of the bioactive inhibitors toward AChE were predicted through the docking study using the MOE-2015.10 software. The data of AChE protein structure was obtained from the Worldwide Protein Data Bank. The most active site (on AChE for docking ligands) was found using the site finder function of the MOE software. Based on the out-put data of MOE, 25 binding sites on AChE were determined. The sizes, residues, and 3D structures of these binding sites were mapped and are presented in the Appendix A (Table A1). The ligands may bind to various sites on the enzyme; however, only the most active site was chosen to be presented and discussed in detail. Based on the site finder function of MOE, and the pre-screening results, binding site 1 is suggested as the most active binding site for further investigation. This active site was found (Figure 3a) to contain 39 residues. To determine whether binding site 1 was covered by the catalytic site of enzyme or not, the CASTp3.0 server was used in the prediction of the AChE catalytic site, which has the volume and the surface area of 904.278 Å^3^ and 529.676 Å^2^, respectively (Figure 3b, Table A2). Figure 3 indicated that binding site 1 was not located in the catalytic site. Thus, all these inhibitors show a high possibility of no binding to the catalytic site of AChE.

In the virtual study, RMSD and DS are important parameters to determine whether a compound (ligand/inhibitor) may bind to the target protein and inhibit its enzymatic activity [51,52]. For a successful binding, when a ligand interacts with a target protein with an RMSD value less than 2.0 Å, the binding is considered to be significant and widely accepted for further virtual analysis [51]. As shown in Table 5, all the identified compounds (**1**–**21**) interacted with AChE with low RMSD values in the range of 0.63–1.75 Å, while the interaction of the commercial inhibitor berberine chloride had an RMSD value of 1.65 Å. This result suggested the successful binding of all the ligands to the target protein with acceptable RMSD values. For virtual evaluation of effective inhibition, DS was commonly used. When a compound binds to the target enzyme with a DS value less than −3.20 kcal/mol, it is proposed as an enzyme inhibitor [52]. In comparison, the lower the DS value an inhibitor has, the greater is its inhibitory effect. As summarized in Table 5, all the compounds could bind to AChE with DS values greater than −6.3 kcal/mol, indicating that they may be possible AChE candidates. The commercial inhibitor berberine chloride showed high inhibition against AChE, with a DS value of −12.1 kcal/mol. Compared with the commercial inhibitor, eleven compounds (**1**–**10**, **20**) exhibited weaker AChE inhibition (DS value in the range of −6.3 to −11.0 kcal/mol) than berberine chloride (**22**). Other compounds (**11**–**19** and **21**) demonstrated higher activity than berberine chloride (**22**) with low DS values of −12.3 to −14.4 kcal/mol. Overall, the order of inhibition of the AChE protein by active compounds was as follows: **17** > **13** > **18** > **16** > **14** > **11** = **19** > **15** > **12** > **21** > **22** (commercial inhibitor) > **20** > **9** > **7** > **1** > **3** > **2** > **8** > **6** > **10** > **4** > **5**. The result of the in vitro test and virtual analysis could nearly fit and corroborate each other, indicating that the meOH extract of ELC trunk bark showed a high effectiveness against AChE, due to harboring various major active compounds (**11**–**19**,**21**).

**Figure 4:**
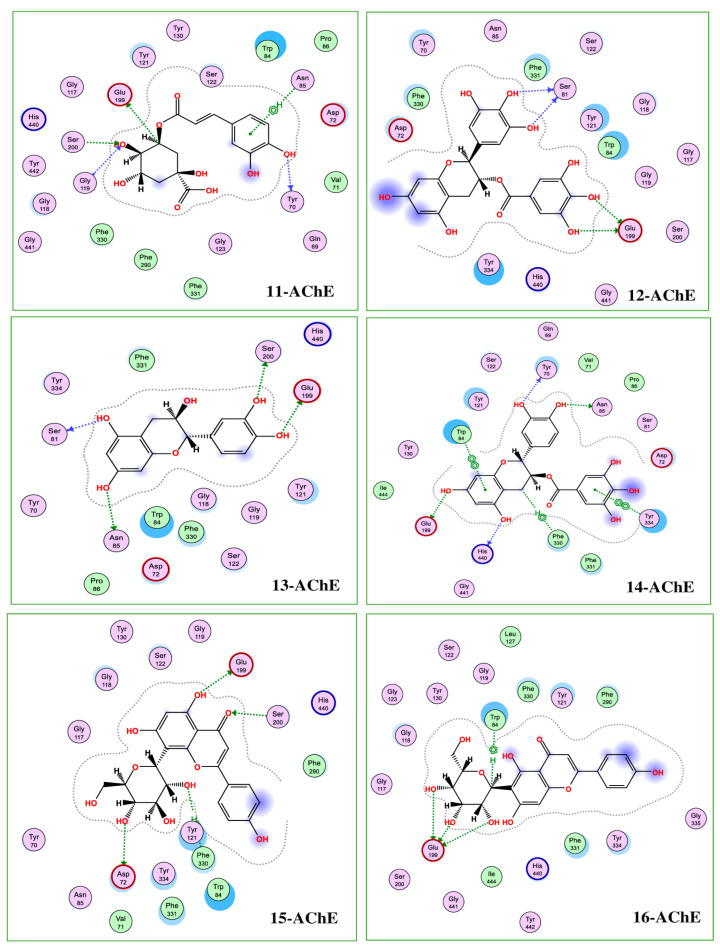

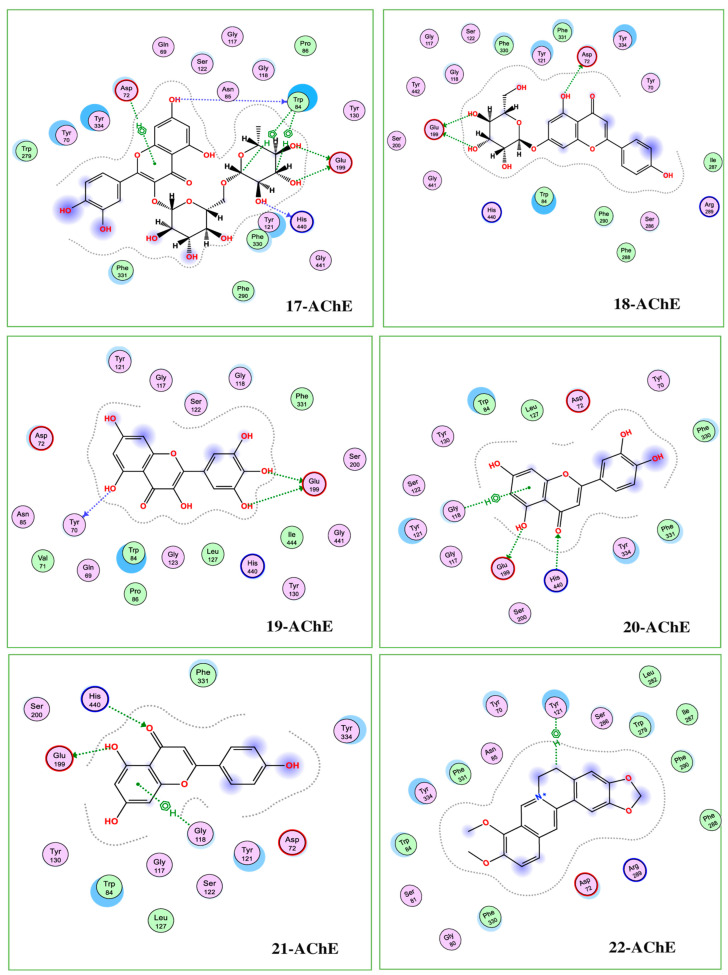
Insight into interactions of ligand–acetylcholinesterase (AChE) at the binding site on AChE (ligand: **11**–**22**).

To investigate the interaction of ligands and AChE, the detailed binding at the active site were recorded, as presented in Table 6 and Figure 4. The active inhibitor compounds (**11**–**19**,**21**) and commercial inhibitor (**22**) were examined. Rutin (**17**) demonstrated the highest inhibition against AChE by interacting with four amino acids, including His400, Glu199, Trp84, and Asp72 of this enzyme, resulting in the creation of seven linkages (4 H-donor, 2 H-pi, 1 pi-H). Among these, this ligand (**17**) was found to interact with His400 and Asp72 and formed one H-donor linkage and one pi-H linkage, respectively, while it interacted with Glu199 and Trp84 to create two linkages (H-donor), and three linkages (2 H-pi, 1 pi-H), respectively. This was followed by epicatechin (**13**), which showed efficient inhibition against AChE at a low DS value (−14.3 kcal/mol) by binding and interacting with this enzyme through four amino acids, Ser81, Asn85, Ser200, and Glu199, to generate four H-donor linkages. The next five compounds (**11**, **14**, **16**, **18**, and **19**) could also bind tightly to AChE with DS values lower than −13.1 kcal/mol by interacting with five, seven, two, two, and two amino acids to form five, seven, four, three, and four linkages, respectively. The binding of apigetrin (**18**) to AChE was through the least interaction (three linkages) but possessed the best binding energy (DS value of −13.9 kcal/mol), while other ligands had greater interaction with AChE (four to seven linkages) but showed weaker bind energies (DS values in the range of −13.1 to −13.8 kcal/mol). These results indicated that the DS values may be independent of the number of interactions between the ligand and the enzyme. The next three ligands (**12**, **15**, and **21**) also showed a slightly higher binding effect to AChE than the commercial inhibitor (**22**). These compounds, **12**, **15**, and **21,** could bind to AChE by interacting with two, four, and three amino acids and generated four, four, and three linkages, respectively, while the commercial inhibitor (**22**) binds to AChE through only one amino acid, forming only one linkage. Among the prominent amino acids contained in the binding site, Glu199 had an important role in the interaction with all the above-mentioned ligands (**11**–**19**, and **21**). This amino acid (Glu199) was bound to the ligands **11**, **13**, **14**, **15**, **20**, and **21** to generate one H-donor linkage (binding linkage energy in the range of −0.6 to −5.8 kcal/mol) with each ligand, while it interacted with ligands **12**, **17**, and **18** to generate two H-donor linkages (binding linkage energy in the range of −0.5 to −3.8 kcal/mol) with each ligand. Especially, Glu199 was found to bind with ligands **16** and **19,** and for each ligand, up to three H-donor linkages (binding linkage energy in the range of −1.1 to −4.0 kcal/mol) were formed.

**Figure 5:**
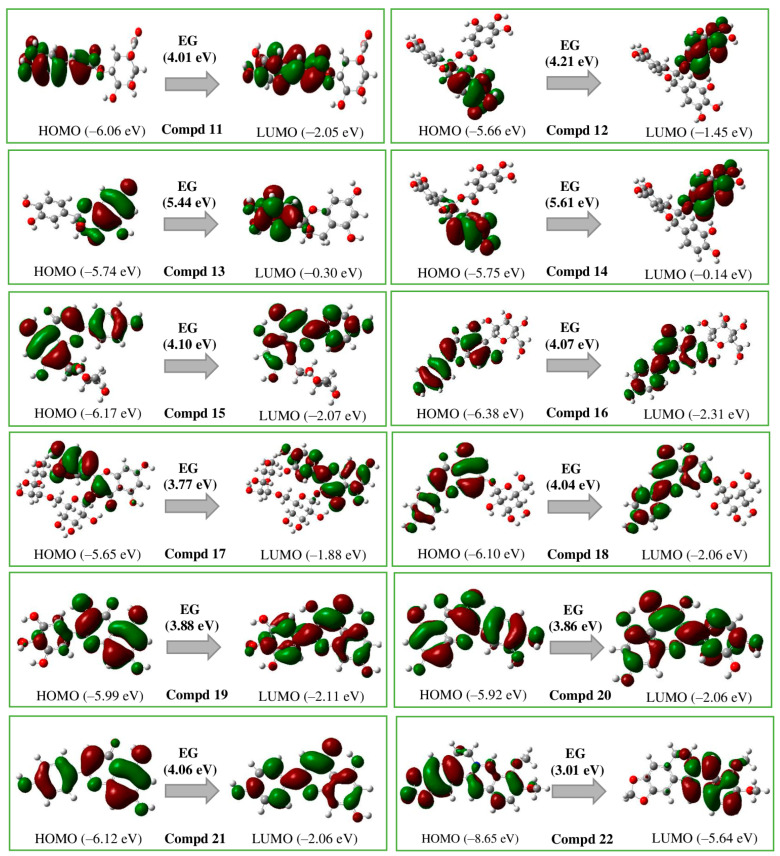
HOMO and LUMO of compounds **11**–**22** analyzed by DFT at level of theory B3LYP/6–31G.

These potential molecules were further investigated in their frontier molecular orbitals. The data of the highest occupied molecular orbital (HOMO) and lowest unoccupied molecular orbital (LUMO) of these compounds are presented in Figure 5. These molecular structures showed their low *E_HOMO_* values in the range of − 5.65 to − 8.65 eV. This indicates their significant electronic stability (commonly accepted under − 5 eV). In the previous investigation [53], theoretical complexes of the tetrylone family with *E_HOMO_* values of − 3 to − 7 eV were also found as highly stable. It has been evidenced that all structures possess an insulation-to-semiconduction energy gap (3.2 eV < EG < 9 eV), showing good intermolecular binding capability toward protein structures [54], and this was further explained based on the super-exchange theory [55,56] and the electron hopping model [57]. In this study, the energy gap values of the inhibitors were found in the range of 3.77 to 5.61 eV; as such, they have potential intermolecular binding capability toward the targeting enzyme.

Based on RMSD, DS values and some data of frontier molecular orbitals recorded, some secondary metabolites (compounds **11**–**19**, **21**) contained in the methanol extract of ELC trunk bark may be suggested as potential candidates of AChE inhibitors. However, some works summarized by Pagadala et al. [58] indicated unreliable binding affinity predictions using docking studies. Several reports also indicated some drawbacks of the docking study [59,60,61]. Thus, further works should be performed, including purifications of active compounds and testing activity via in vitro, in vivo, and clinical trials for development of these compounds to be drugs.

### 3.4. Lipinski’s Rule of Five and ADMET-Based Pharmacokinetics and Pharmacology

Lipinski’s rules have been applied to evaluate the drug-likeness of compounds; the five rules include “molecular mass must be less than 500 Da (rule 1), high lipophilicity with LogP value < 5 (rule 2), hydrogen bond donors <5 (rule 3), hydrogen bond acceptors <10 (rule 4), and the molar refractivity should be between 40–130 (rule 5)”. A compound is considered to have drug-likeness properties and has a high possibility of being a drug when it satisfies at least 2/5 of Lipinski’s rules. As presented in Table 7, all the identified compounds complied with five of Lipinski’s rules, except compound **2,** which complied with four of Lipinski’s rules. Thus, these compounds have a high probability of successfully being developed as a drug.

The ADMET properties of these compounds and commercial inhibitors were also compared, and the data are presented in Appendix A (Figure A3 and Figure A4). In general, these compounds in the MeOH extract of ELCTB also showed good ADMET properties in the required allotted limitation. In addition, all the active inhibitor compounds (**11**–**19** and **21**) and the commercial inhibitor (**22**) were not toxic for human use. Furthermore, the evidence of safety on normal cells of some compounds was indicated via in vitro or in vivo tests. Chlorogenic acid was recorded to affect cancer cells without normal cell influence [62]. EGCG was found as toxic on HuCC-T1 cancer cells but safe on the viability of 293T normal cells [63]. In another report, EGCG from green tea at 40–200 microM caused a significant death for some tumor cell lines, but only 1% of the WI38 normal cells growth was affected in the same condition [64]. Epicatechin and Epicatechin gallate were discovered to cause death for various cancer cells but had no effect on normal cells in some reports [65,66,67,68]. Vitexin has a non-effect on bronchial epithelial 16HBE normal cells [69]. Rutin, Apigetrin, and Myricetin all are potential and safe adjuvant chemotherapeutic agents with trivial toxicity and side effects via in vivo and clinical trials [70,71,72]. The scientific proof also demonstrated that Quercetin and Apigenin have no or low effects on normal cells [71,73]. Almost phenolics in this study were showed safe for normal cells. However, the safe evidence for all compounds detected still needs to be performed continually in further research via in vitro, in vivo, and clinical trials.

## 4. Conclusions

This is the first report of AChE inhibition of the MeOH extract of ELCTB. From this extract, 21 secondary metabolites (**1**–**21**) were identified, including 10 volatile compounds (**1**–**10**), 1 phenolic (**11**), and 10 flavonoids (**12**–**21**). Of these, compounds **1**–**11, 15**, **16**, **17**, **18**, **19**, **20**, and **21** were detected for the first time in this herbal extract. Compounds (**11**–**19** and **21**) exhibited greater effective inhibitory activity than the commercial inhibitor with good binding energy and acceptable RMSD values. The Lipinski rule of five and ADMET analyses indicated that the identified compounds possessed drug properties and were non-toxic for human use. The finding of this study indicates that ELC is a rich source of bioactive compounds that have the potential to be used as anti-Alzheimer drug candidates.

## Figures and Tables

**Table 1:** Acetylcholinesterase inhibitory effect of ELC trunk bark extracted by various solvents.

No.	ELCTB Extracted by Various Solvents	IC_50_ (mg/mL)	Max Inhibition (%)
**1**	Methanol extract	0.323 ± 0.021	97.0 ± 2.3%
**2**	Butanol extract	0.781 ± 0.042	80.1 ± 2.4%
**3**	Ethyl acetate extract	1.232 ± 0.098	65.2 ± 3.1%
**4**	*n*-Hexane extract	1.530 ± 0.101	57.3 ± 1.5%
**5**	Water extract	0.662 ± 0.032	79.1 ± 2.6%
**6**	Berberine chloride	0.314 ± 0.032	98.2 ± 1.8%

**Table 2:** Acetylcholinesterase-inhibitory activity, the total content of polyphenol, and flavonoid of different parts of *Euonymus laxiflorus* Champ.

Parts Used of *Euonymus laxiflorus* Champ. Extracted by MeOH	IC_50_ (mg/mL)	Total Content of Polyphenol (mg GAE/g Dry Extracts)	Total Content of Flavonoid (mg QE/g Dry Extracts)
Heartwood MeOH extract	1.326 ± 0.103	107.2 ± 22.1	98.0 ± 7.8
Trunk bark MeOH extract	0.336 ± 0.029	567.3 ± 27.2	335.2 ± 10
Leaves MeOH extract	0.981 ± 0.045	227.2 ± 15.1	101.3 ± 8.9
Berberine chloride	0.327 ± 0.031	-	-

**Table 3:** The volatile compounds in the MeOH extract of ELC trunk bark identified by GCMS analysis.

ID Compd	Compds	RT (min)	% Area
**1**	3-Hydroxydecanoic acid	3.40	48.63
**2**	Propane, 1,1-dipropoxy- (CAS)	4.77	2.91
**3**	(2-(2-butoxyisopropoxy)-2-isopropanol	4.88	2.62
**4**	*p*-Xylene	5.16	11.63
**5**	Styrene	5.56	8.53
**6**	Oxalic acid, heptyl propyl ester	7.45	4.62
**7**	Sulfurous acid, isobutyl pentyl ester	9.19	2.58
**8**	2-Phenylethyl allyl ether	10.28	0.38
**9**	12-Hydroxyalliacolide	12.76	8.04
**10**	7-ethyl-Quinoline	13.91	5.92

**Table 4:** The content of phenolic compounds contained in the methanol extract of ELC trunk bark by UPLC.

ID Compd	Compds	RT (min)	Content μg/g of Dried Extract
**11**	Chlorogenic acid	13.700	395.808
**12**	EGCG	14.198	402.680
**13**	Epicatechin	14.760	576.809
**14**	Epicatechin gallate	16.230	79.513
**15**	Vitexin	17.785	23.197
**16**	Isovitexin	19.180	63.884
**17**	Rutin	19.603	61.581
**18**	Apigetrin	20.442	2481.525
**19**	Myricetin	20.882	221.843
**20**	Quercetin	22.830	487.600
**21**	Apigenin	24.772	184.798

**Table 5:** The RMSD and DS values of ligands (L) binding with AChE.

Compounds (Ligands)	Symbols of L-AChE	RMSD (Å)	DS (kcal/mol)
3-Hydroxydecanoic acid	1-AChE	0.82	−10.1
Propane, 1,1-dipropoxy-(CAS)	2-AChE	0.63	−9.6
(2-(2-butoxyisopropoxy)−2-isopropanol	3-AChE	1.72	−9.8
*p*-Xylene	4-AChE	1.57	−6.5
Styrene	5-AChE	0.93	−6.3
Oxalic acid, heptyl propyl ester	6-AChE	1.11	−8.8
Sulfurous acid, isobutyl pentyl ester	7-AChE	0.81	−10.3
2-Phenylethyl allyl ether	8-AChE	1.68	−8.9
12-Hydroxyalliacolide	9-AChE	1.40	−10.6
7-ethyl-Quinoline	10-AChE	0.82	−8.6
Chlorogenic acid	11-AChE	1.50	−13.1
EGCG	12-AChE	1.24	−12.5
Epicatechin	13-AChE	1.75	−14.3
Epicatechin gallate	14-AChE	1.52	−13.3
Vitexin	15-AChE	1.18	−12.8
Isovitexin	16-AChE	1.09	−13.8
Rutin	17-AChE	1.29	−14.4
Apigetrin	18-AChE	1.03	−13.9
Myricetin	19-AChE	0.77	−13.1
Quercetin	20-AChE	1.51	−11.0
Apigenin	21-AChE	1.26	−12.3
Berberine chloride	22-AChE	1.65	−12.1

**Table 6:** The docking results of ligands (L) binding with acetylcholinesterase (AChE).

L-AChE Complex	Linkages Number	Amino Acids Interacting with the Ligands [Distance (Å)/E (kcal/mol)/Linkage Type]
11-AChE	5 linkages (2 H-donor, 2 H-acceptor, 1 pi-H)	Tyr70 (2.75/−2.5/H-donor); Glu199 (3.42/−0.6/H-donor); Gly119 (2.78/1.6/H-acceptor); Ser200 (2.68/−1.2/H-acceptor); Asn85 (4.66/−0.6/pi-H)
12-AChE	4 linkages of H-donor	Ser81 (2.79/−3.7/H-donor); Ser81 (3.18/−2.6/H-donor); Glu199 (3.60/−0.5/H-donor); Glu199 (2.8/−5.0/H-donor)
13-AChE	4 linkages of H-donor	Ser81 (2.77/−2.8/H-donor); Asn85 (3.10/−1.0/H-donor); Ser200 (2.78/5.6/H-donor); Glu199 (2.99/−2.1/H-donor)
14-AChE	7 linkages (4 H-donor, 1 H-pi, 2 pi-pi)	His440 (2.59/−1.5/H-donor); Glu199 (2.87/−2.7/H-donor); Tyr70 (2.60/−2.5/H-donor); Asn85 (2.56/−1.9/H-donor); Phe330 (3.91/−1.1/H-pi); Trp84 (3.92/−0.0/pi-pi); Tyr334 (3.74/−0.0/pi-pi)
15-AChE	4 linkages (2 H-donor, 1 H-acceptor, 1 H-pi)	Asp72 (3.29/−1.0/H-donor); Glu199 (2.84/−4.3/H-donor); Ser200 (2.62/−1.1/H-acceptor); Phe330 (4.32/−1.3/H-pi)
16-AChE	4 linkages (3 H-donor, 1 H-pi)	Glu199 (3.17/−1.1/H-donor); Glu199 (2.61/−3.5/H-donor); Glu199 (2.89/−4.0/H-donor); Trp84 (3.55/−0.8/H-pi)
17-AChE	7 linkages (4 H-donor, 2 H-pi, 1 pi-H)	His400 (2.75/−2.2/H-donor); Glu199 (3.00/−2.0/H-donor); Glu199 (2.90/−3.0/H-donor); Trp84 (3.31/−0.9/H-donor); Trp84 (4.02/−0.9/H-pi); Trp84 (4.10/−0.6/H-pi); Asp72 (3.57/−0.8/pi-H)
18-AChE	3 linkages of H-donor	Glu199 (3.04/−2.7/H-donor); Glu199 (2.92/−3.8/H-donor); Asp72 (2.88/−1.5/H-donor)
19-AChE	4 linkages of H-donor	Tyr70 (2.98/−2.1/H-donor); Glu199 (2.81/−1.4/H-donor); Glu199 (2.80/−2.1/H-donor); Glu199 (2.84/−1.5/H-donor)
20-AChE	3 linkages (1 H-donor, 1 H-acceptor, 1 pi-H)	Glu199 (2.75/−5.8/H-donor); His440 (3.02/−1.5/H-acceptor); Gly118 (3.77/−0.7/pi-H)
21-AChE	3 linkages (1 H-donor, 1 H-acceptor, 1 pi-H)	Glu199 (2.76/−5.8/H-donor); His440 (3.04/−1.1/H-acceptor); Gly118 (3.74/−0.7/pi-H)
22-AChE	1 H-pi	Tyr121 (4.50/−0.7/H-pi)

**Table 7:** The result of Lipinski’s Rule of Five of the compounds identified in the methanol extract of ELC trunk bark (**1**–**21**), and berberine chloride (**22**).

ID Compd	Mass (Dalton)	Hydrogen Bond Donor	Hydrogen Bond Acceptors	LogP	Molar Refractivity
**1**	187.0	1	3	0.848	49.01
**2**	160.0	0	2	22.576	46.56
**3**	190.0	1	3	2.284	52.32
**4**	106.0	0	0	2.303	35.92
**5**	104.0	0	0	2.33	36.53
**6**	230.0	0	4	2.45	60.97
**7**	209.0	1	3	2.787	56.26
**8**	162.0	0	1	2.432	51.16
**9**	187.0	1	3	0.848	49.01
**10**	157.0	0	1	2.797	51.210
**11**	157.0	0	1	2.797	51.121
**12**	353.0	5	9	−1.981	79.89
**13**	458.0	8	11	2.233	108.92
**14**	290.0	5	6	1.546	72.62
**15**	442.0	7	10	2.528	107.26
**16**	432.0	7	10	−0.066	103.53
**17**	432.0	7	10	−0.066	103.53
**18**	610.0	10	16	−1.879	137.50
**19**	432.0	6	10	−0.107	103.54
**20**	318.0	6	8	1.717	75.72
**21**	302.0	5	7	2.011	74.05
**22**	270.0	3	5	2.420	70.81
Lipinski’s rules	≤500	≤5	≤10	≤5	40–130

**Figure A5:**
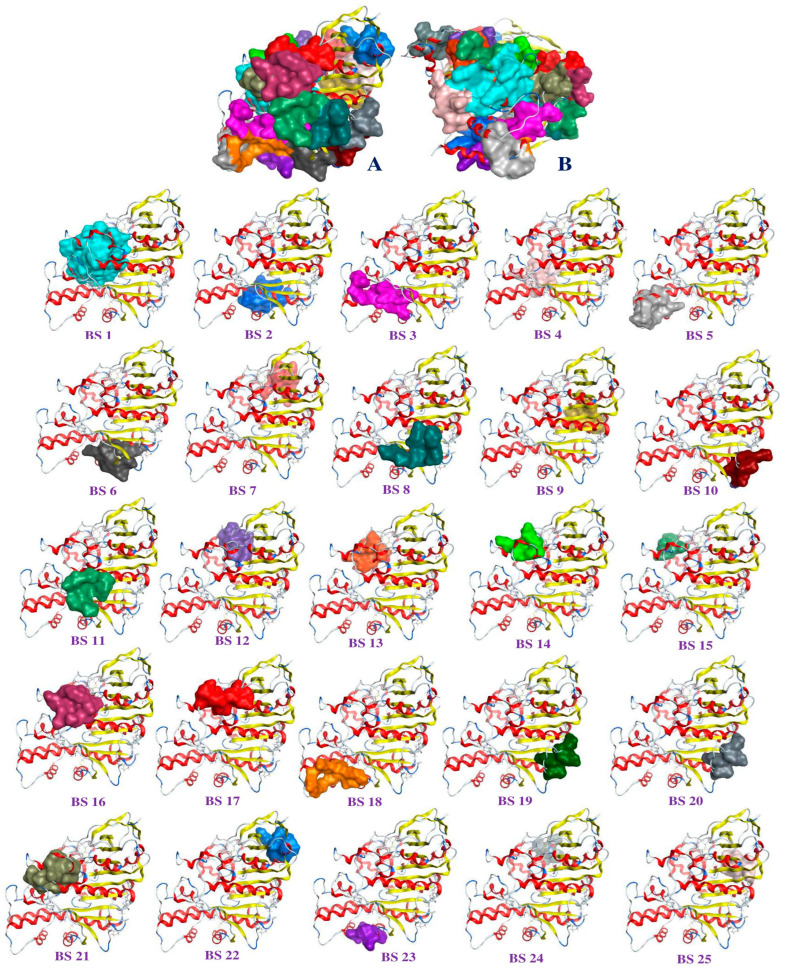
Mapping the binding sites on AChE. The 3D structure of binding sites from the front surface (**A**) and back surface (**B**) of AChE. The 3D structure of 25 binding sites on AChE (named **1**–**25**).

## Data Availability

All the data for this article can be found in the article.

## Appendix A

**Table A1:** The detail data of catalytic site prediction of extracellular domain of AChE found via using the CASTp3.0 server.

Name of Protein	Surface Area (SA) Å^2^	Volume (SA) Å^3^
*Electrophorus electricus* AChE (1EEA)	529.676	904.278
Amino acids located at the active site of 1EEA: PRO229 ASN230 CYS231 PRO232 TRP233 SER 235 VAL236 SER237 GLU240 ARG244 LEU282 PRO283 PHE284 SER286 ARG289 PHE290 VAL293 ILE296 SER304 LEU305 GLU306 PRO361 HIS362 HIS398 CYS402 PRO403 HIS406 TRP524 ASN525 GLN526 LEU 528 PRO529 LEU532 ASN533		

**Table A2:** The detail size and residues of 25 binding sites of acetylcholinesterase was found using MOE-2015.10.

SITE	SIZE	Residues
1	202	1:(GLN69 TYR70 VAL71 ASP72 GLN74 SER81 TRP84 ASN85 PRO86 TYR116 GLY117 GLY118 GLY119 TYR121 SER122 GLY123 SER124 LEU127 TYR130 GLU199 SER200 TRP233 TRP279 LEU282 PHE284 ASP285 SER286 ILE287 PHE288 ARG289 PHE290 PHE330 PHE331 TYR334 GLY335 HIS440 GLY441 TYR442 ILE444)
2	39	1:(ASN230 PRO232 GLU306 ASP397 HIS398 CYS402 PRO403 HIS406 TRP524 ASN525 PRO529)
3	32	1:(LYS325 ASP326 ARG388 ASP389 ASP392 ASP393 ILE401 PHE422 GLU434 TRP435 ARG517)
4	20	1:(PRO232 GLU240 ARG244 LEU282 PRO283 PHE284 ASP285 SER286 ARG289 PRO361 HIS362)
5	20	1:(ARG349 PHE352 TYR375 THR376 ASP377 ASP380 ASP381 LYS386 ASN387 GLY390 LEU391)
6	27	1:(CYS402 MET405 HIS406 ASN409 LYS410 GLN500 ARG515 LEU516 ARG517 VAL518 CYS521 VAL522 ASN525 GLN526)
7	19	1:(ALA36 GLU37 PRO38 PRO39 MET43 ARG46 ARG47 PRO48 GLU49 LEU95 ARG149)
8	17	1:(ARG468 TYR472 SER487 GLU489 SER490 GLU508 PRO509 MET510)
9	22	1:(ARG47 GLY166 ASN167 LEU171 ARG174 SER212 PRO213 GLY214 ASP297 GLU299 PHE300)
10	19	1:(THR412 GLY415 ASN416 GLY417 THR418 LEU494 PHE495 THR496 THR497)
11	16	1:(GLU461 ALA464 LEU465 ARG468 ASN506 THR507 GLU508 PRO509)
12	24	1:(PRO39 ARG44 CYS67 GLN68 SER91 GLU92 CYS94 TYR148 ARG149 VAL150 PHE153)
13	12	1:(GLN68 GLN69 TYR70 TYR121 VAL150 GLY151 ALA152 PHE153 LEU274 ILE275 GLU278)
14	18	1:(GLU82 MET83 ASN85 ASN87 LEU127 ASP128 VAL129)
15	17	1:(GLN69 TYR70 VAL71 GLN272 ILE275 ASP276)
16	19	1:(LEU450 PRO451 LEU452 VAL453 LYS454 LEU456 ASN457 TYR458 THR459 ALA460 GLU463)
17	23	1:(ASP128 VAL129 ASN131 LYS133 TYR134 LEU450 VAL453 GLU455 LEU456)
18	20	1:(TYR375 THR376 ASP377 LYS386 ASP389 GLY390 ASP393 ARG517 MET520)
19	12	1:(LEU31 GLY32 TRP58 ASN59 ALA60 SER61 THR62 TYR63 PRO64)
20	21	1:(GLN318 ASN416 GLY417 TYR419 THR479 GLY480 ASN481 LEU494)
21	41	1:(MET83 VAL129 ASN429 LEU430 VAL431 TYR442 GLU445 LEU450 LEU456 TYR458)
22	7	1:(LYS11 SER12 LYS51 TRP179 ASP182 ASN183)
23	12	1:(GLN374 GLN519 MET520 VAL522 PHE523 PHE527)
24	17	1:(ASN42 GLU163 GLU260 ILE263 HIS264 ARG267)
25	16	1:(ARG47 PRO48 LEU171 ARG174 MET175 GLN178 LEU218)

**Table A3:** The absorption, distribution, metabolism, excretion, and toxicity (ADMET)-based pharmacokinetics and pharmacology of identified compounds contained in the methanol extract of *Euonymus laxiflorus* Champ. trunk bark (**1**–**11**).

ID Compd	1	2	3	4	5	6	7	8	9	10	11	Unit
Property												
**Absorption**												
Water solubility	−2.327	−2.051	−1.454	−2.522	−2.739	−2.3	−2.3	−2.633	−2.777	−2.445	−2.449	(1)
Caco2 permeability	1.398	1.653	1.726	1.547	1.544	1.45	1.755	1.563	0.336	1.619	−0.84	(2)
Intestinal absorption (human)	93.346	95.839	93.25	95.713	95.902	95.192	93.991	97.018	78.829	97.318	36.377	(3)
Skin Permeability	−2.713	−2.144	−3.472	−1.236	−1.104	−2.082	−2.018	−1.505	−3.257	−1.642	−2.735	(4)
P-glycoprotein substrate	No	No	No	No	No	No	No	No	No	Yes	Yes	(5)
P-glycoprotein I inhibitor	No	No	No	No	No	No	No	No	No	No	No	(5)
P-glycoprotein II inhibitor	No	No	No	No	No	No	No	No	No	No	No	(5)
**Distribution**												
VDss (human)	−0.799	0.054	−0.075	0.325	0.403	−0.034	−0.046	0.414	0.206	0.184	0.581	(6)
Fraction unbound (human)	0.454	0.55	0.603	0.362	0.331	0.454	0.489	0.284	0.622	0.27	0.658	(6)
BBB permeability	0.252	0.63	0.005	0.409	0.459	0.555	0.525	0.688	−0.008	0.396	−1.407	(7)
CNS permeability	−2.92	−2.69	−3.083	−1.677	−1.577	−2.849	−2.331	−1.884	−3.367	−1.92	−3.856	(8)
**Metabolism**												
CYP2D6 substrate	No	No	No	No	No	No	No	No	No	No	No	(5)
CYP3A4 substrate	No	No	No	No	No	No	No	No	No	No	No	(5)
CYP1A2 inhibitor	No	No	No	No	Yes	No	No	Yes	No	Yes	No	(5)
CYP2C19 inhibitor	No	No	No	No	No	No	No	No	No	Yes	No	(5)
CYP2C9 inhibitor	No	No	No	No	No	No	No	No	No	No	No	(5)
CYP2D6 inhibitor	No	No	No	No	No	No	No	No	No	No	No	(5)
CYP3A4 inhibitor	No	No	No	No	No	No	No	No	No	No	No	(5)
**Excretion**												
Total Clearance	1.598	1.639	1.35	0.254	0.265	1.774	0.535	0.35	0.803	0.296	0.307	(9)
Renal OCT2 substrate	No	No	No	No	No	No	No	No	No	No	No	(5)
**Toxicity**												
AMES toxicity	No	No	No	No	No	No	No	No	Yes	Yes	No	(5)
Max. tolerated dose (human)	0.084	0.796	0.848	0.921	0.943	0.771	0.805	0.999	0.529	0.331	−0.134	(10)
hERG I inhibitor	No	No	No	No	No	No	No	No	No	No	No	(5)
hERG II inhibitor	No	No	No	No	No	No	No	No	No	No	No	(5)
Oral Rat Acute Toxicity (LD50)	1.342	2.004	1.949	1.841	1.83	1.947	2.189	1.828	3.097	2.023	1.973	(11)
Oral Rat Chronic Toxicity	2.552	2.269	2.105	2.168	2.225	2.087	2.043	1.946	1.901	2.11	2.982	(12)
Hepatotoxicity	No	No	No	No	No	No	No	No	No	No	No	(5)
Skin Sensitization	No	Yes	Yes	No	No	Yes	Yes	Yes	No	No	No	(5)
T.Pyriformis toxicity	0.38	0.596	0.181	−0.022	−0.018	0.163	0.833	0.96	0.31	0.453	0.285	(13)
Minnow toxicity	0.711	1.336	1.956	1.31	1.136	0.477	0.854	0.67	2.574	0.643	5.741	(14)

Note: **^(1)^** log mol·L^−1^; **^(2)^** log Papp (10^−6^ cm·s^−1^); **^(3)^** %; **^(4)^** log Kp; **^(5)^** Yes/No; **^(6)^** log L·kg^−1^; **^(7)^** log BB; **^(8)^** log PS; **^(9)^** log mL·min^−1^·kg^−1^; **^(10)^** log mg·kg^−1^·day^−1^; **^(11)^** mol·kg^−1^; **^(12)^** log mg·kg^−1^_bw·day^−1^; **^(13)^** log μg·L^−1^; **^(14)^** log mM.

**Table A4:** The absorption, distribution, metabolism, excretion, and toxicity (ADMET)-based pharmacokinetics and pharmacology of identified compounds contained in the methanol extract of *Euonymus laxiflorus* Champ. trunk bark (**12**–**21**) and berberine chloride (**22**).

ID Compd	12	13	14	15	16	17	18	19	20	21	22	Unit
Property												
**Absorption**												
Water solubility	−2.894	−3.117	−2.911	−2.845	−2.812	−2.892	−2.559	−2.915	−2.925	−3.329	−6.715	(1)
Caco2 permeability	−1.521	−0.283	−1.264	−0.956	−0.618	−0.949	0.33	0.095	−0.229	1.007	1.212	(2)
Intestinal absorption (human)	47.395	68.829	62.096	46.695	64.729	23.446	37.609	65.93	77.207	93.25	94.642	(3)
Skin Permeability	−2.735	−2.735	−2.735	−2.735	−2.735	−2.735	−2.735	−2.735	−2.735	−2.735	−2.781	(4)
P-glycoprotein substrate	Yes	Yes	Yes	Yes	Yes	Yes	Yes	Yes	Yes	Yes	No	(5)
P-glycoprotein I inhibitor	No	No	No	No	No	No	No	No	No	No	Yes	(5)
P-glycoprotein II inhibitor	Yes	No	Yes	No	No	No	No	No	No	No	Yes	(5)
**Distribution**												
VDss (human)	0.806	1.027	0.664	1.071	1.239	1.663	0.342	1.317	1.559	0.822	0.179	(6)
Fraction unbound (human)	0.215	0.235	0.158	0.242	0.21	0.187	0.218	0.238	0.206	0.147	0	(6)
BBB permeability	−2.184	−1.054	−1.847	−1.449	−1.375	−1.899	−1.391	−1.493	−1.098	−0.734	0.764	(7)
CNS permeability	−3.96	−3.298	−3.743	−3.834	−3.754	−5.178	−3.746	−3.709	−3.065	−2.061	−1.652	(8)
**Metabolism**												
CYP2D6 substrate	No	No	No	No	No	No	No	No	No	No	No	(5)
CYP3A4 substrate	No	No	No	No	No	No	No	No	No	No	Yes	(5)
CYP1A2 inhibitor	No	No	No	No	No	No	No	Yes	Yes	Yes	No	(5)
CYP2C19 inhibitor	No	No	No	No	No	No	No	No	No	Yes	No	(5)
CYP2C9 inhibitor	No	No	No	No	No	No	No	No	No	No	No	(5)
CYP2D6 inhibitor	No	No	No	No	No	No	No	No	No	No	No	(5)
CYP3A4 inhibitor	Yes	No	No	No	No	No	No	No	No	No	No	(5)
**Excretion**												
Total Clearance	0.292	0.183	−0.169	0.444	0.442	−0.369	0.547	0.422	0.407	0.566	0.619	(9)
Renal OCT2 substrate	No	No	No	No	No	No	No	No	No	No	No	(5)
**Toxicity**												
AMES toxicity	No	No	No	No	No	No	No	No	No	No	No	(5)
Max. tolerated dose (human)	0.441	0.438	0.449	0.577	0.649	0.452	0.515	0.51	0.499	0.328	−0.653	(10)
hERG I inhibitor	No	No	No	No	No	No	No	No	No	No	No	(5)
hERG II inhibitor	Yes	No	Yes	No	No	Yes	No	No	No	No	Yes	(5)
Oral Rat Acute Toxicity (LD50)	2.522	2.428	2.558	2.595	2.558	2.491	2.595	2.497	2.471	2.45	2.553	(11)
Oral Rat Chronic Toxicity	3.065	2.5	2.777	4.635	5.37	3.673	4.359	2.718	2.612	2.298	0.89	(12)
Hepatotoxicity	No	No	No	No	No	No	No	No	No	No	No	(5)
Skin Sensitization	No	No	No	No	No	No	No	No	No	No	No	(5)
T.Pyriformis toxicity	0.285	0.347	0.285	0.285	0.285	0.285	0.285	0.286	0.288	0.38	0.432	(13)
Minnow toxicity	7.713	3.585	6.146	4.897	5.18	7.677	5.507	5.023	3.721	2.432	−1.711	(14)

Note: **^(1)^** log mol·L^−1^; **^(2)^** log Papp (10^−6^ cm·s^−1^); **^(3)^** %; **^(4)^** log Kp; **^(5)^** Yes/No; **^(6)^** log L·kg^−1^; **^(7)^** log BB; **^(8)^** log PS; **^(9)^** log mL·min^−1^·kg^−1^; **^(10)^** log mg·kg^−1^·day^−1^; **^(11)^** mol·kg^−1^; **^(12)^** log mg·kg^−1^_bw·day^−1^; **^(13)^** log μg·L^−1^; **^(14)^** log mM.
